# A cohort study of *in utero *polychlorinated biphenyl (PCB) exposures in relation to secondary sex ratio

**DOI:** 10.1186/1476-069X-7-37

**Published:** 2008-07-15

**Authors:** Irva Hertz-Picciotto, Todd A Jusko, Eric J Willman, Rebecca J Baker, Jean A Keller, Stuart W Teplin, M Judith Charles

**Affiliations:** 1 Department of Public Health Sciences, School of Medicine, TB #168, University of California-Davis, Davis, CA, 95616, USA; 2 Department of Epidemiology, School of Public Health and Community Medicine, University of Washington, Box 357236, Seattle, WA, 98195, USA; 3 Department of Environmental Toxicology, University of California-Davis, Davis, CA, 95616, USA; 4 Ecolab, Eagen, MN, 55121, USA; 5 Department of Epidemiology, School of Public Health, University of North Carolina-Chapel Hill, Chapel Hill, NC, 27599, USA; 6 Quintiles, Inc, 5927 South Miami Blvd, Morrisville, NC, 27560, USA; 7 Center for the Study of Development and Learning, Department of Pediatrics, University of North Carolina-Chapel Hill School of Medicine, Chapel Hill, NC, 27599, USA

## Abstract

**Background:**

Polychlorinated biphenyls (PCBs) are ubiquitous industrial chemicals that persist in the environment and in human fatty tissue. PCBs are related to a class of compounds known as dioxins, specifically 2,3,7,8-TCDD (tetrachloro-dibenzodioxin), which has been implicated as a cause of altered sex ratio, especially in relation to paternal exposures.

**Methods:**

In the 1960's, serum specimens were collected from pregnant women participating in the Child Health and Development Study in the San Francisco Bay Area. The women were interviewed and their serum samples stored at -20°C. For this study, samples were thawed and a total of eleven PCBs were determined in 399 specimens. Secondary sex ratio, or sex ratio at birth, was evaluated as a function of maternal serum concentrations using log-binomial and logistic regression, controlling for hormonally active medications taken during pregnancy.

**Results:**

The relative risk of a male birth decreased by 33% comparing women at the 90^th ^percentile of total PCBs with women at the 10^th ^percentile (RR = 0.67; 95% CI, 0.48–0.94; p = 0.02), or by approximately 7% for each 1 μg/L increase in total PCB concentration. Although some congener-specific associations with sex ratio were only marginally statistically significant, all nine PCB congeners with < 30% of samples below the LOQ showed the same direction of association, an improbable finding under the null hypothesis.

**Conclusion:**

Maternal exposure to PCBs may be detrimental to the success of male sperm or to the survival of male embryos. Findings could be due to contaminants, metabolites or PCBs themselves.

## Background

Polychlorinated biphenyls (PCBs) are a class of chemically stable compounds that were produced in the United States from the 1920's until 1977, and in Europe beginning in the 1940's or 1950's for several decades, and were widely used in industrial and some consumer products. Each individual PCB congener represents one configuration of 209 that are possible, based on the number and location of attached chlorine atoms. These compounds bioaccumulate up the food chain, such that the greatest human sources are meat, fish, and both animal and human breast milk. Because of their high lipophilicity, PCBs are not readily cleared from the body [1-3].

A number of organochlorines, their metabolites, and their contaminants have been characterized as endocrine-disrupting chemicals. PCBs are structurally similar to thyroxine, which is critical to *in utero *development [2-4]. Animal studies have demonstrated altered thyroid homeostasis following PCB exposure, although human studies have produced mixed results [5]. Additionally, PCB congeners, their metabolites, or related compounds (e.g., 2,3,7,8-tetrachlorodibenzo-*p*-dioxin and dichloro-diphenyl-trichloroethane (DDT)) have been shown to have estrogenic, anti-estrogenic or anti-androgenic activity [6-9], and some human studies have found reduced sperm motility among PCB-exposed subjects [10-12].

Following the chemical explosion that resulted in dioxin contamination of residential areas in Seveso, Italy, a deficit in male births of couples with high exposures was observed [13,14]. A similar finding was reported for the offspring of over 2,000 adults exposed in 1979 to cooking oil in Taiwan that was contaminated with a mix of PCBs, polychlorinated dibenzo furans and polychlorinated dibenzo dioxins [15]. Data from a similar accident in Japan were not consistent with an alteration in sex ratio [16], but this report included a relatively small number of births. Few studies at lower environmental exposure levels have reported on sex ratio, and most have not used direct measurements of individual PCBs.

This paper examines sex ratio in births occurring between 1964 and 1967 in the San Francisco Bay Area in relation to concentrations of serum polychlorinated biphenyls in archived serum specimens that were collected during the pregnancies. The participants were a subset of those enrolled in the Child Health and Development Study (CHDS), a cohort of about 20,000 pregnancies. Because the CHDS was conducted during the 1960's, a time of peak production and use of these compounds, body burdens were higher than those found in recent general population studies in western countries [17,18], yet lower than some current highly-exposed populations [19,20].

## Methods

### Study population

The CHDS was a prospective cohort study that enrolled about 20,000 pregnant women either attending prenatal clinics or giving birth at Kaiser Foundation Health Plan Medical Centers in the San Francisco Bay Area during the 1960's [20-22]. The Kaiser Health Plan, a prepaid integrated health care system, at that time had enrolled 90 percent of membership through employer or union groups (G Friedman, Kaiser-Permanente, personal communication,1996). The population served was thus largely persons having stable employment, with wealthy and very poor persons generally not well represented. Otherwise, members represented a broad cross-section of the San Francisco Bay Area population.

The 399 woman/child pairs in this study were selected from 3,412 CHDS children born April 1964 through April 1967 who participated in an extensive examination at five years of age, described previously [23,24]. To be eligible for our study (Table 1), women had to have completed the enrollment interview before delivery and donated a second or third trimester blood specimen, for which a sufficient volume of serum still remained. As the study was designed to examine a range of birth and developmental outcomes, exclusions were made for: 1) non-singleton deliveries, 2) children with severe anomalies or who did not complete two cognitive exams and a hearing screening; 3) mothers who were deaf, had rubella during pregnancy, were taking thyroid medication in the 60 days prior to blood draw, or took iodine-containing medications during pregnancy or in the six months prior to conception [25]; and 4) infants born at gestational ages that were unknown, less than 35, or greater than 45 completed weeks. Based on regularly updated contact information provided by the CHDS investigators, we selected children residing in eight counties in or surrounding the greater San Francisco Bay Area. The study was approved by the Human Subjects Review Board at the University of California, Davis.

**Table 1:** Exclusions of CHDS children from a 5-Year examination subset for organochlorine study

		Excluded		
Step	Reason for exclusion	N	%	Cases remaining

1	Mother is an unmarried minor or no interview completed	587	17.2	2825
2	Blood was drawn prior to second trimester or after 3^rd ^trimester	177	5.2	2648
3	Not a singleton birth	28	0.8	2620
4	Raven or Peabody cognitive test score missing	60	1.8	2560
5	Hearing tests were not done in both ears	146	4.3	2414
6	Confounding anomalies present	21	0.6	2393
7	Mother is deaf	2	0.1	2391
8	Congenital rubella*	46	1.3	2345
9	Mother took a drug containing iodine	86	2.5	2259
10	Thyroid drugs were taken within 60 d of blood draw	7	0.2	2252
11	Gestational age is less than 244 or greater than 351 days	99	2.9	2153
12	No current address is available	468	13.7	1685
13	Does not reside in 1 of 8 included counties	337	9.9	1348
14	Only one sibling in family can be included; other siblings dropped	57	1.7	1291

*Included any mention of rubella in the chart, whether or not a diagnosis was made.

After these exclusions, there remained a group of 1,291 children. We sampled all children with low scores on at least one of two cognitive tests (below 10^th ^percentile on either the Peabody Picture Vocabulary Test or an abbreviated Raven's Progressive Matrices), or who failed a hearing screening, and a 17 percent random sample of all others, to achieve the targeted sample size. The motivation for this design was to enhance power for the hypotheses related to neurocognitive and neurosensory deficits (which were of relatively low prevalence) while not adversely affecting efficiency for other hypotheses. Several authors have published papers on "reusing" data from case- control studies – designs that are, in essence, a special case of stratified sampling [26,27]. At the analysis stage, we adjusted for this design by applying normalized weights in all regression model fitting (detailed below). Finally, if women had more than one eligible child, we sampled no more than one child for each mother. Serum PCB concentrations were determined for 399 women in this final sample.

### PCB measurement

Laboratory methods have been described previously [17,28]. Briefly, serum specimens collected during pregnancy were aliquoted and stored at -20° Celsius at National Institutes of Health (NIH) facilities. For determination of PCBs the protocol was as follows: Samples were thawed and then homogenized; surrogate standards (e.g, 2,3,4,4'-tetrachorobiphenyl (PCB International Union of Pure and Applied Chemistry (IUPAC) #66)), and 2,3,3',5,5',6-hexachlorobiphenyl (PCB #165) and glacial acetic acid were added, and the solution was mixed. The PCBs were extracted three times with 90 percent hexane/10 percent dichloromethane, and the extract was passed through a 0.5 percent deactivated florisil column. The PCBs were eluted with 60 milliliters of hexane and then 60 milliliters of 50 percent hexane/50 percent dichloromethane. After concentration by rotary evaporation, the internal standard 2,2',3,4,4',5,6,6'-octachlorobiphenyl (PCB #204) was added. Serum extracts were analyzed by gas chromatography with electron capture detection (Hewlett-Packard 6890 Series) in parallel using an RTX-5MS and an RTX-1701 column. All samples were analyzed between January 14, 1998 and September 22, 1999 in batches of 10 to 20 samples. Concentrations were adjusted for percent recovery for each sample. Specimens analyzed on batch dates with extremely high or low variability were re-analyzed, and the two values averaged.

Before analyzing any samples from our subjects, noncritical samples from the same cohort (i.e. of women in CHDS who were lost to follow-up) were analyzed to assess the integrity of the stored serum specimens. Organochlorine determinations demonstrated comparability with other historical samples, and lipids were in reference ranges.

We included PCBs that: (a) were measured in mixtures reported (as of the late 1990's) to have associations with reproductive, developmental or endocrine alterations in animals or humans; (b) did not co-elute with other compounds on either of the two columns; and (c) were present in sufficient concentration in these samples [28]. These included: PCB #101, #105, #110, #118, #137, #138, #153, #156, #170, #180, and #187. Values below the limit of quantitation (LOQ) were imputed as described previously [17]. The total of PCBs was calculated as the sum of the nine congeners that had fewer than 30% below the LOQ (PCB IUPAC #105, #110, #118, #137, #138, #153, #170, #180, and #187).

### Outcome and other variables

Sex of the infant was collected at the time of birth and noted in the CHDS database. Because of our design, only data on pregnancies that resulted in a live birth were included.

Potential covariates were selected from CHDS databases which included data on sociodemographics and maternal health and pregnancy. To adjust for the potential effects of maternal sex hormone or corticosteroid use during the periconception period, all prescriptions and associated dates were extracted for women in this study. A complete list of medications prescribed during the index period between thirty days prior to and four months after conception was reviewed using references pertinent for the time period of the study [29-31]. Active ingredients in medications from earlier years were checked against current references [32,33]. Mothers were coded (yes/no) according to whether they had been prescribed sex hormones or corticosteroids. Other potential adjustment variables for the multivariate analysis were specimen characteristics. These were: batch-date of laboratory analysis and an indicator for sample storage history (some specimens had been immediately shipped for storage at an NIH facility while others had been stored locally and later shipped).

### Data analysis

Log-binomial and logistic regression analyses were conducted to estimate the relative risk (RR) and odds ratio (OR), respectively, of male birth as a function of maternal PCB concentrations. Since the odds ratio is a poor approximation of the relative risk when the outcome is common [34], the relative risk of male birth was estimated using log-binomial regression [35], and forms the basis of our primary analysis. As a secondary analysis and for purposes of comparison with previous research, we estimated the odds of male birth in relation to maternal PCB concentrations using logistic regression. All regression models were adjusted for specimen characteristics and prescription for sex hormones or corticosteroids. Some maternal factors have been implicated with altered sex ratio such as maternal age, parity, and race [36]. However, since inclusion of these measures did not alter PCB coefficients by more than 10 percent, they were not included in our final multivariate models. Separate models were fit for total PCBs and for the 9 specific PCB congeners that had less than 30 percent of their values below the LOQ. In all regression models, PCB concentration was modeled as a continuous variable in micrograms per liter of serum (μg/L) (parts per billion (ppb)). To provide a more interpretable measure of the PCB "effect size," we calculated a risk ratio comparing sex ratio at the 90^th ^vs. 10^th ^percentiles of PCBs by multiplying the per-unit PCB coefficient by the concentration difference between the 90^th ^and 10^th ^percentiles. In addition, normalized weights were applied to adjust for our stratified sampling procedure; these weights were inversely proportional to the sampling fraction in each stratum. When applied to the regression analysis, the results are as if the sample of 399 children was drawn completely at random from the subset of 1291 children. Thus, any potential bias created by the stratified sampling procedure is removed. As in standard survey research, this procedure ensures that all parameter estimates are valid with respect to the larger sample or population from which the stratified sample has been selected, and eliminates any bias introduced by use of a stratified sample. To account for potentially greater homogeneity within sampling strata, robust variance estimates were calculated for both log-binomial and logistic regression models. All analyses were conducted using the GENMOD procedure in SAS (version 9.1; SAS Institute, Inc., Cary, North Carolina).

## Results

### Sample characteristics

Table 2 compares the sociodemographic characteristics of the original CHDS cohort, the 3,412 children who appeared for the 5-year examination, the 1,291 eligible after exclusions, and the final study sample of 399. As compared with the original cohort, the mothers of those examined at five years were of similar age, more highly educated, and less likely to be white. Those eligible and selected for our study differed from the original cohort on the same factors, with a greater percentage of African-Americans meeting our eligibility criteria. Female children were slightly more prevalent among those participating in the 5-year examination than in the original cohort, and among those eligible after our exclusions; finally, more females were included in our study because of poorer average performance on the Raven's Progressive Matrices, one of the cognitive tests for which children with low scores were over-sampled. Our study sample included 53.4 percent females as compared with 48.8 percent of the original cohort. As described above, normalized weights were used to account for stratified sampling; since the sampling altered the sex ratio, these weights also resulted in correction for the differential selection by gender.

**Table 2:** Sample characteristics, Child Health and Development Study, 1964–67

	Final stratified random sample (n = 399)		Cases meeting study criteria (n = 1291)		Cases followed up at 5 years (n = 3412)		CHDS study population (n = 20754)	
Characteristics*	N	%†	N	%	N	%	N	%

Maternal age								
< 20	28	7	68	5	230	7	1731	9
20–29	242	61	786	61	2099	62	12028	59
≥ 30	128	32	436	34	1068	31	6724	33
Parity								
0	122	31	400	31	1077	32	6289	31
1–2	177	44	605	47	1572	47	9270	45
≥ 3	100	25	286	22	717	21	5031	24
Race/ethnicity								
White	194	49	648	50	1970	58	1347	66
African-American	165	41	496	38	1041	31	4936	24
Hispanic	7	2	40	3	130	4	678	3
Asian	20	5	70	5	150	4	783	4
Multi-racial/other	13	3	37	3	108	3	597	3
Mother's education								
Not High School graduate	76	19	208	16	500	17	3341	19
High School graduate	160	40	484	38	1118	37	6661	38
Some college	163	41	599	46	1378	46	7549	43
Father's education								
Not High School graduate	74	19	211	20	496	17	2997	18
High School graduate	111	29	339	32	789	27	4607	28
Some college	122	32	431	41	979	34	4329	26
College grad/RN	76	20	286	27	643	22	4681	28
Sex of infant								
Female	213	53.4	658	51.0	1722	50.5	9451	48.8
Male	186	46.6	633	49.0	1690	49.5	9927	51.2

*Data missing for some characteristics for some study participants.

†Percentages may not total 100% due to rounding.

### PCB concentrations

The mean, median, 10^th ^and 90^th ^percentile of total maternal PCBs were: 5.4, 4.7, 3.1, and 8.7 μg/L (ppb) of serum, respectively. PCB #153, the most abundant congener in this study, had a median of 1.1 μg/L (10^th ^percentile: 0.6 μg/L serum; 90^th ^percentile: 1.9 μg/L serum). These PCB concentrations are similar to levels reported by others for the United States from the same time period [37], and higher than most but not all populations under study today [18].

### PCB concentrations and secondary sex ratio

Log-binomial regression analyses, in which we adjusted for specimen characteristics and hormonally active medications, revealed that total maternal PCB concentration was associated with a reduction in male births (p = 0.02). Specifically, the relative risk of a male birth decreased by 33 percent comparing women at the 90^th ^percentile of total PCBs with those at the 10^th ^percentile of total PCBs (RR = 0.67; 95% CI: 0.48, 0.94), or by approximately 7 percent for each 1 μg/L (1 ppb) increase in total PCBs (Table 3). In log-binomial regression models for specific PCB congeners, the predicted relative risk of a male birth decreased with higher maternal PCB concentration in each and every congener measured, though most congeners were only marginally associated with male birth. The results from logistic regression models were similar in direction, showing, as expected, larger odds ratios than the relative risks derived from the log-binomial regression analyses.

**Table 3:** Adjusted relative risk (RR) and odds ratio (OR) for a male birth (n = 399)*

	Log-binomial model			Logistic model		
PCB exposure	RR	95% CI	p-value	OR	95% CI	p-value

Total PCBs†	0.67	0.48, 0.94	0.02	0.45	0.26, 0.80	0.007
PCB 105	0.76	0.57, 1.01	0.06	0.58	0.37, 0.91	0.02
PCB 110	0.79	0.59, 1.06	0.11	0.66	0.41, 1.07	0.09
PCB 118	0.85	0.65, 1.12	0.25	0.72	0.45, 1.15	0.17
PCB 137	0.77	0.57, 1.04	0.09	0.63	0.39, 1.02	0.06
PCB 138	0.82	0.62, 1.09	0.17	0.68	0.42, 1.09	0.10
PCB 153	0.82	0.61, 1.11	0.20	0.68	0.40, 1.15	0.15
PCB 170	0.75	0.56, 1.00	0.05	0.57	0.36, 0.91	0.02
PCB 180	0.89	0.69, 1.14	0.35	0.80	0.51, 1.24	0.32
PCB 187	0.80	0.58, 1.09	0.16	0.67	0.38, 1.17	0.16

*Adjusted RR and OR for a male birth for a given increase in PCB concentration from the 10^th ^to the 90^th ^percentile: Child Health and Development Study, 1964–67. Estimates adjusted for specimen characteristics and an indicator of a prescription for sex steroids, oral contraceptives or corticosteroids.

†Sum of PCB #105, 110, 118, 137, 138, 153, 170, 180, 187.

## Discussion

We observed a reduction in the ratio of male:female births in relation to total maternal PCB concentrations in this population. Inverse associations between all nine PCB congeners and secondary sex ratio were also observed, though not all of these were statistically significant at the 0.05 level. In models adjusted for hormonal medications taken in the periconceptional and early pregnancy period, higher concentrations of total PCBs were significantly associated with male birth ratio (p = 0.02). As PCB blood concentrations have been shown to remain very stable during pregnancy [37], these findings suggest that high maternal PCB concentrations, at least for some congeners, may either favor the fertilization by female sperm, or result in greater male embryonic or fetal losses. Sex ratio alterations or decreased survival of wildlife in association with organochlorine exposures have been well documented [38-41], although not all studies have confirmed such findings [42].

Only a handful of human studies of PCBs with sufficient sample sizes have reported data on sex ratio. At environmental exposures, several provide some suggestion of a reduced proportion of males [43-46]. Weisskopf and colleagues [46] examined consumers of sport-fish caught in the Great Lakes and observed a reduction in male births in association with maternal but not paternal serum PCB concentration. In contrast, another investigation found increased male offspring in relation to paternal PCB exposures, though this analysis may have over-adjusted for multiple exposures correlated with paternal PCBs [47]. Reports from the Yucheng [48] and Yusho [16] accidents showed no alteration in sex ratio, however, serious shortcomings in these studies should be noted. The Yucheng group consisted of a select set of mothers who had registered with the health department as having been affected, and who had at least one live child in 1985. Not all affected subjects were registered, and some of the most severely affected children may not have survived: since either of these factors could have been related to the sex of the child, the sex ratio of the survivors could be highly confounded. Moreover, both the Yucheng and the Yusho studies were small: a total of 137 births in the former and only 67 in the latter. These sample sizes would not be adequate to detect small variations in sex ratio. Analysis of a more complete cohort from Yucheng, numbering 469 births with paternal data and 902 with maternal data revealed reduced male:female sex ratio when fathers were exposed at a young age [15], results similar to those previously reported for dioxin exposures from the Seveso accident. Analyses for maternal exposures also showed a lower than expected proportion of males, but the findings were not statistically significant. The difference in findings using maternal vs. paternal exposure appeared to be due, at least partly, to a higher percentage of male offspring among unexposed fathers than among unexposed mothers, or, than expected in the population. Since every child has both a mother and a father, but fewer fathers were tested, this raises the question of selection bias for children whose fathers participated in the study.

In addition to studies of secondary sex ratio, research on the proportion of Y-bearing spermatozoa in relation to paternal PCBs have produced mixed results, i.e., both positive and negative associations [49,50]. This would suggest that increased paternal PCB concentrations may also alter secondary sex ratio, but other factors may modify this effect. It is not clear from these studies – due to inadequate sample size – whether changes in Y-bearing spermatozoa resulted in more or fewer male births.

The possibility of bias deserves consideration. Selection factors would have to be associated with sex ratio and PCBs, jointly to have produced artifacts for the relationship of these variables of primary interest. Since demographic variables are not generally associated with sex ratio, they would not bias the results. The slight preponderance of girls followed up at age 5 years (50.5%) as compared to the original cohort (48.8% female), combined with our deliberate oversampling of children with low cognitive scores or hearing difficulties increased the percentage of females to 53.4%, however, our sampling weights accounted for these differences, comparable to their standard use in survey sampling. It is possible that because a small number of deaths occurred between birth and five years of age, part of the shift in sex ratio might be due to far higher deaths among boys with higher PCBs, or among girls with lower PCBs. At these exposure levels, however, which were far lower than occurred in the Yusho or Yucheng incidents, it seems unlikely that PCBs would have had a sufficiently strong association with child mortality to produce the results observed and yet not be related to fetal mortality. If PCBs were strongly associated with unknown or gestational age below 35 weeks, some selection bias could have occurred by our exclusion of these births. However, the literature does not support an association of preterm delivery with PCBs, nor did we find any trend of higher PCBs with earlier gestational age among births at weeks 35–45 [23]. In regard to other variables, the sample was representative of the original cohort. Thus, selection bias seems unlikely to be of sufficient magnitude to explain the findings.

Validity of our data is supported by the confirmation of known risk factors for other outcomes, such as maternal smoking, short stature, and low body mass index in relation to intrauterine growth restriction [23]. High quality PCB determinations were ensured by use of within-batch replicates, standards across batches, and surrogate standards. Thus, in comparison with previous work, our study is unique in that it thoroughly addresses maternal exposures, uses an adequate sample, has high quality direct measurements of PCB exposures close in time to the events of interest, and represents exposures at the upper end of today's environmentally exposed populations.

## Conclusion

In this study, we observed a lower secondary male:female ratio in association with increased maternal serum PCB concentrations. If not due to an unidentified bias, these results may indicate that with higher exposures to PCBs comes either greater susceptibility of male embryos or a more favorable environment for X-chromatin-bearing sperm. The association could be due to contaminants, PCB metabolites, or the PCBs themselves.

Although PCB concentrations are slowly declining, studies in historical populations such as the CHDS provide an upper limit on the magnitude of toxic effects expected from today's environmental levels of these same compounds in most parts of the world. Secondly, these investigations are useful for assessing reproductive perturbations in populations currently exposed to higher levels, e.g., those that rely on fish from contaminated lakes and seas or reside near former manufacturing facilities. Thirdly, if the findings are actually due to contaminants associated with PCB mixtures rather than PCBs themselves, then attention should be paid to trends in the contaminants, regardless of their source. Finally, other chemical classes with similar structure, such as PBDEs (polybrominated diphenyl ethers), are widely used in plastic casings and foam products and share many of the biochemical and toxicologic properties of PCBs [51,52]. As PBDE body burdens are rapidly increasing in wildlife and human populations [53,54], studies like this one provide an indication of potential effects from these newer compounds.

## Competing interests

The authors declare that they have no competing interests.

## Authors' contributions

IHP was PI of this project, communicated with collaborators, designed and supervised the statistical analyses, and had primary responsibility for writing the manuscript. TJ conducted log-binomial and logistic analyses, with adjustment for sampling and robust variance estimators and helped edit the manuscript. MJC ran the laboratory which conducted the PCB analyses, participated in designing the study, in preparing manuscripts, and in study meetings until her death in September 2005. EW worked under MJC and carried out the analytic chemical analysis by GC/ECD and performed the quality control work. RJB was a graduate student who assisted in writing the grant proposal, cleaning and managing the data, and preliminary statistical analysis. She also participated actively in discussions of early results, prior to her death. JK provided key programming support for development and management of all the databases and quality control, and participated in study meetings and development of the analysis plans during the first three years of the study. ST provided expertise on early childhood development, assisted with study design and exclusion criteria, and gave guidance on medical conditions and medications used as covariates for study sample restriction and for adjustment in the analyses. IHP, TJ, EW, JK, and ST read and approved the final manuscript.
